# Video feedback and e-Learning enhances laboratory skills and engagement in medical laboratory science students

**DOI:** 10.1186/s12909-019-1745-1

**Published:** 2019-08-14

**Authors:** Rebecca Donkin, Elizabeth Askew, Hollie Stevenson

**Affiliations:** School of Health and Sport Sciences, University of the Sunshine Coast, 90 Sippy Downs Drive, Sippy Downs, 4556 Queensland, Australia

**Keywords:** Medical laboratory science, Blended learning, Interactive learning environments, Media in education, Simulations

## Abstract

**Background:**

Traditionally, the training of medical laboratory science students has taken place in the laboratory and has been led by academic and pathology experts in a face-to-face context. In recent years, budgetary pressures, increasing student enrolments and limited access to laboratory equipment have resulted in reduced staff-student contact hours in medical laboratory science education. While this restructure in resources has been challenging, it has encouraged innovation in online blended learning.

**Methods:**

Blended learning histology lessons were implemented in a face-to-face and e-Learning format in a medical laboratory science program to teach tissue morphology and technical procedures outside of the traditional laboratory classroom. Participating students were randomly allocated to either the ‘video’ group (*n* = 14) or the ‘control’ group (*n* = 14). After all students attempted the e-Learning lessons and viewed expert-led video recordings online, students demonstrated their hands-on practical skills in the laboratory. Technical skills, demonstration of safety awareness, and use of histology equipment was captured by video through first person ‘point of view’ recordings for the ‘video’ group only. The ‘control’ group performed the same activities but were not recorded. Prior to summative assessment, the ‘video’ group students had a digital resource portfolio that enabled them to review their skills, receive captured feedback and retain a visual copy of their recorded procedure.

**Results:**

Results showed that students who participated in the online video format had statistically better practical examination scores and final grades compared to the control group.

**Conclusion:**

Findings from this study suggest that students are engaged and motivated when being taught in a blended learning format and respond positively to the use of video recordings with expert feedback for the initial learning of hands-on techniques. For the academic, developing a blended learning medical laboratory science program, which includes annotated virtual microscopy, video demonstrations, and online interactive e-Learning activities, provides an effective and economic approach to learning and teaching.

## Background

### Introduction

Medical laboratory science (MLS) programs that develop curricula with evidence-based practice, critical thinking, research and scholarship, report that student enthusiasm and motivation are key elements to positive learning outcomes [1, 2]. Previously, this enthusiasm has been engendered through laboratory-based study, rather than in the lecture theatre using books and passive learning. Blended learning, using a combination of face-to-face and online activities, has proven to be comparable with or even improve the grades achieved by the traditional teaching of MLS [3, 4].

Blended learning facilitates learning in a variety of physical places and at different times. In addition, the student controls the pace and mode of the teaching and learning experience which may consist of: recorded lectures; interactive online modules; web based learning; virtual or simulated learning, mobile device learning (tablet or phone), learning management systems, e-Learning; and learning platforms facilitated through asynchronous internet tools [5, 6].

Previously, providing resources for students to learn outside of the laboratory involved (often poor quality) video recordings, postings of self-made clips on websites (which may be in unrelatable settings or produced for non-related disciplines), or online activities which fail to simulate the technique for a learner [5]. However, successful course design and the associated materials must have a student-centred approach to increase student engagement and to improve learning outcomes [7].

One such method of blended learning is the use of video assisted feedback to capture a ‘point of view’ recording of the student’s attempt to perform a laboratory procedure. Video recording is not a new technology, it requires minimal operative knowledge and the end product does not require high cognitive load or skills in information literacy to extract and assess the learning objective. The recording allows the student to receive feedback and improve skills while also providing a comparison between how the learner perceives their performance to that given by the expert [8].

Although video assisted feedback of practical skills is not new, there is limited evidence [9] that video recording of the student skills and technical ability when in the pathology laboratory increases engagement with learning and improves grade scores. The aim of our study was to determine if students enrolled in a MLS program displayed greater engagement with the subject and achieved higher grade results when using video feedback and online resources.

### Educational context

Students enrolled in a MLS program in 2017, were invited to participate in the study. The participants were studying a first-year undergraduate course in histology that ran for 13 weeks which explored general pathology: structure and function of cells, tissue and organs of the human body, and the technical aspects used in pathology services, including preparation of tissue samples for light microscopy. At the end of the course students were required to complete a practical examination to demonstrate their skills in laboratory techniques and morphological identification. For successful completion of the course students were required to attend a minimum of 80% of the laboratory practicals and achieve an overall 50% grade or higher in the practical examination.

The practical examination was assessed by two experts in histology. Students independently performed histology techniques which included: embedding- orienting and mounting samples into paraffin wax; and microtomy- sectioning tissue samples on a microtome. Throughout these exercises the students were viewed by the assessor who marked the technique on the basis of: safety- hazard avoidance; knowledge of techniques and use of equipment; confidence in performing the procedures without reliance on notes; knowledge as to why and how the technique was performed; and the quality of the final product.

To supplement their learning, students were provided with several online resources that formed a digital learning portfolio that did not require laboratory access. The portfolio included: online web based resources and student activities (quizzes, short answer questions, annotated morphology pictures); 24-h access to virtual slides (e.g. annotated moveable online tissue sections); online tutorial feedback provided by an expert in histology; interactive online learning modules (e-Learning); and expert-led video demonstrations in the histology laboratory.

## Methods

### Course delivery

The histology course had run successfully (as measured by final grade and the university’s graduate attributes) for the previous 4 years (2013–2016) however, due to increased student enrolment, reduced staffing, a decreased equipment budget, and a strategic priority to implement a blended learning curriculum, the course was reviewed and restructured. The learning content remained the same however the delivery was different. Due to the reduction in face-to-face contact hours, major online initiatives were implemented that included online only delivery of lecture material and an online student portfolio that contained: online video recordings of histology techniques (recordings of experts and students); and an online e-Learning module (Introduction to Histology). All students had access to the expert histology technique video demonstrations and online modules from week 6 of the semester to reinforce their learning. Table 1 illustrates the changes from the former (pre-2017) conventional curriculum to the current (2017) curriculum which integrates blended learning tools for course delivery.

**Table 1:** Curriculum renewal summary pre-and post-online interventions of blended learning tools

Teaching Component	Former Course Delivery	Current Course Delivery
Lecture	Face-to-face and online 2 h/week for 13 weeks	Online only 2 h/week for 13 weeks
Tutorial	Large class, didactic, independent learning (+/− computer access) 1 h/week for 13 weeks	Team-based learning (4–6/group), with computer access 3 h/fortnight for 13 weeks
Practical	2 h/week for 13 weeks	2 h/week for 13 weeks
Virtual slides	Available	Available
Video feedback^a^	Not available	Available online for embedding and microtomy techniques
Online e-Learning modules	Not available	Available for histology techniques
Online student portfolio	Not available	Available for morphology and histology techniques (embedding and microtomy)
Face-to-face contact hours	65 h/ semester	44 h/ semester

^a^Available to video study participants in 2017 only

**Fig. 1:**
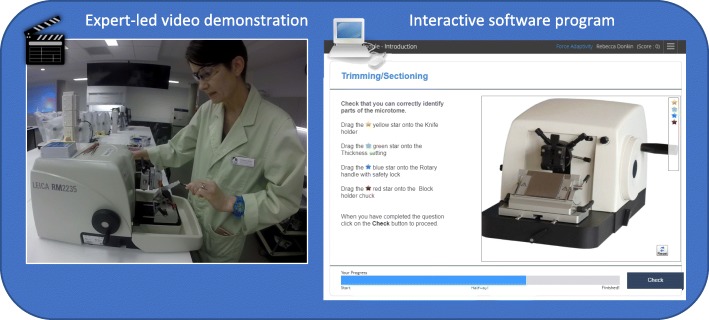
Example of a virtual histology lesson created through an interactive software platform that allows the user to view an expert-led demonstration of a histology technique, then choose the level of engagement and interactive feedback through a ‘drop and drag’ simulated activity. The learner can interact with the activity by using the cursor to drag the appropriate star to a region of interest on the microtome and then receive instant feedback regarding their choices. After three incorrect attempts, the module highlights the correct answer with feedback and directed learning, providing the learner with a choice to proceed or review further material. Analytics are available to the academic to monitor each question and answer and adapt the question or activity to suit the learner

The online e-Learning module was developed in The SmartSparrow Adaptive e-Learning Platform [10] and consisted of interactive and engaging activities to learn histology techniques that could be completed in 20–30 min. The module incorporated embedded videos recorded on a GoPro© or an iPhone 6 s© to capture: first person ‘point of view’ technical skills of an expert performing the histology technique; adaptive feedback; ‘drop and drag’ morphology; multiple choice questions; ‘roll over’ and annotated answers; extended feedback from a virtual ‘Biomedical Scientist’; and student-led learning (choice of activities). Figure 1 illustrates an example of an online module that included an expert led video demonstration and an interactive ‘drop and drag’ activity for a histology technique (microtomy) with feedback and capacity to complete multiple attempts.

### Study design

The study involved a mixed methods approach of quantitative and qualitative methods. Students were assigned with a non-identifiable code number in week 4 of the semester and randomly allocated to one of two groups, ‘video’ or ‘control’ to assess student skills, knowledge and experience in histology techniques delivered in an online ‘video’ and face-to-face ‘control’ format.

**Fig. 2:**
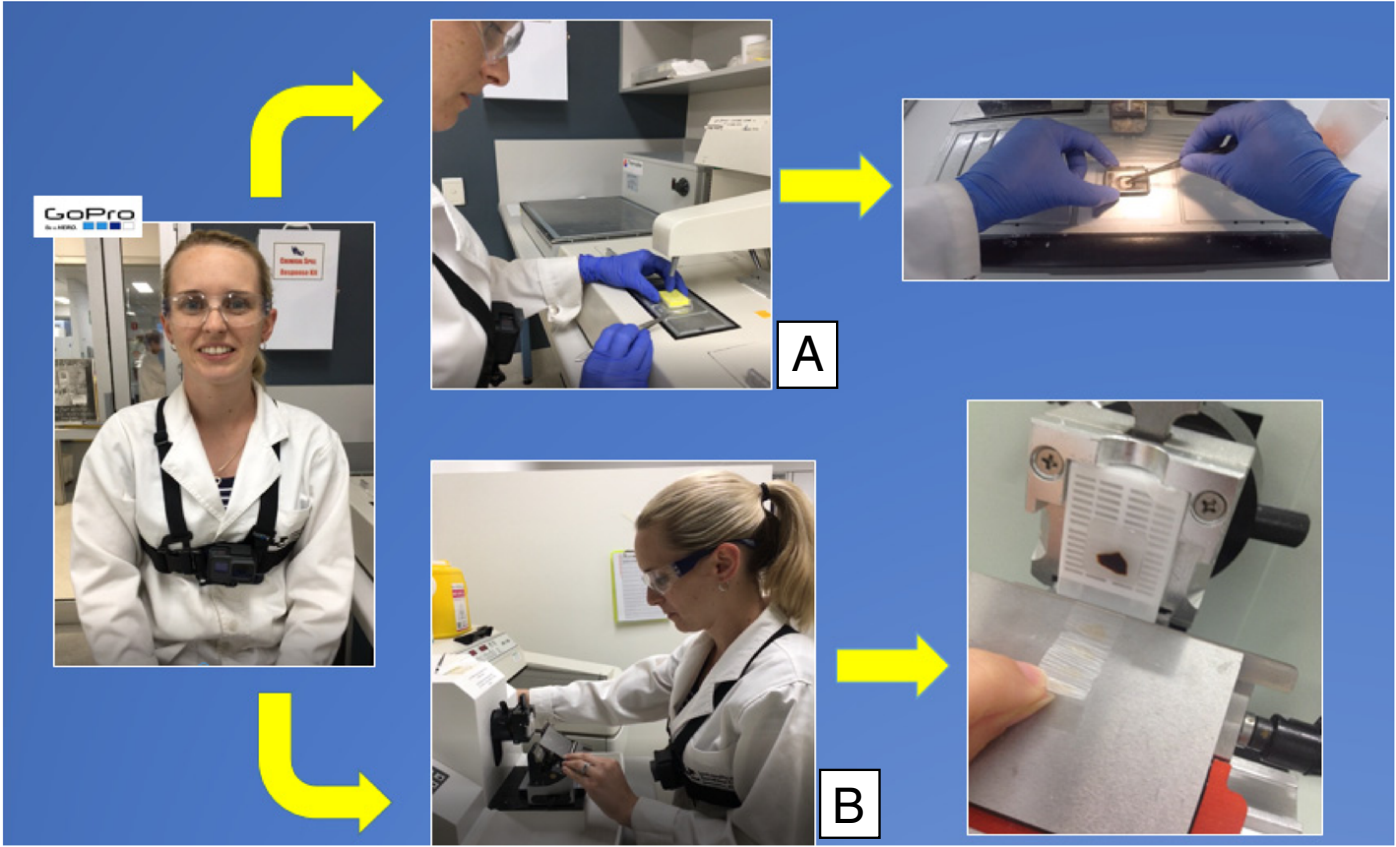
Example of a chest mounted GoPro© recording a first person ‘point of view’ histology technical skill in **a** embedding and **b** microtomy

All students had three formative attempts at the histology techniques from weeks 6–9 before a summative assessment in week 11. In the first and second attempts all students were supervised by two histology Instructors and received individual oral and written (rubric) formative feedback on technical skills, safety and advice for improvement. On the third formative attempt students in the ‘video’ group used a chest mounted GoPro© and tri-pod recording through an iPhone 6 s© to capture first person ‘point of view’ technical skills (Fig. 2). The recording was made by the student and supervised by a student peer, not by an Instructor. Students did not receive oral feedback by the student peer or Instructor at the time of the recording. The video did not contain any identifiable features of the student such as face, hands, or skin. The file was saved with the non-identifiable code number and made available through the student’s online portfolio using the university learning management system, Blackboard™. The video recording could be viewed multiple times by the student through a password protected login.

In weeks 7–10 of the semester, after the student had viewed the recordings by themselves, the Instructor completed a blind written assessment (rubric) of the recordings. When all video assessments were completed, the code numbers were released to identify the students and they were invited to participate in a 5 min, one-on-one review of the video with the assessor. Feedback was provided by the assessor in regard to the student’s technical skills, suggestions for improvement, and an opportunity to raise questions about their performance and self-reflect on their technical ability.

Students in the ‘control’ group received the same content delivery in-class and online but were not video recorded performing their final attempt at the histology techniques. The control group received oral feedback from a student peer at the time of the technique (approximately for 5 min for each attempt) and peer written (rubric) feedback.

At the end of the semester, students completed a summative practical examination consisting of activities in histological techniques (including embedding and microtomy) for routine preparation of cells and tissue for compound light microscopy as well as morphological identification of cells, tissues and organs of the human body.

Following the summative assessment students were invited to complete a brief survey to provide details of their experience in learning histology procedures through video recordings (either their own or the expert recordings). The survey collected data using a 5 -point Likert scale (1 = strongly disagree; 2 = disagree; 3 = unsure; 4 = agree; 5 = strongly agree) and open-ended questions in response to: (i) the experience of video tools in a practical course; (ii) recording histology techniques; (iii) receiving video feedback; and (iv) the use of video recordings for self-reflection/self-perception of laboratory skills.

### Method of analysis

Demographics including gender and age were collected as well as who and how often the students participated in online and in-class activities. An attendance register was kept for all laboratory and tutorial classes and at the end of the laboratory practical an Instructor would confirm if the tasks for the lesson had or had not been satisfactorily completed.

The online resources: an introduction to the histology laboratory; expert histology techniques videos (embedding and microtomy demonstrations); and online module analytics were captured through the learning management system, Blackboard™ and SmartSparrow [10]. To exclude any bias due to changes in academic ability or skill level the 2017 pre-test, summative practical assessment and final grade results were compared with the 2016 cohort. The pre-test quiz consisted of multiple choice and short answer questions relating to histology techniques in the laboratory.

### Statistical analysis

Quantitative data from the practical assessment tasks and pre-test quiz were analysed using the Statistical Package for Social Sciences (SPSS) version 22 (SPSS Inc., Chicago, IL). Data were analysed using independent sample t-tests with criteria of a 95% confidence interval and a null hypothesis that there is no significant change in student’s grade following participation in video feedback. A *P* value < 0.05 was a significant level to suggest strong evidence against the null hypothesis. This project was approved by the University of the Sunshine Coast Human Research Ethics Committee A17982.

### Power and sample size

To adequately detect a 15% difference in grade outcomes between student learning in the ‘video’ group versus the ‘control’ group with statistical power 80%, alpha = 0.05 and assuming a standard deviation on 12 points, a sample size of at least 11 students per group was required.

## Results

### Descriptive results

There were 31 students enrolled in MLS121 Histology in 2017 of which 28 agreed to participate in the study. Students were randomly allocated to either the ‘video’ group (*n* = 14; 11 females, 3 males) or the ‘control’ group (*n* = 14; 10 females, 4 males) by week 4. There were 75% females and 25% males, 65% were more than 20 years old and all students were enrolled in a MLS program. These descriptive statistics were an accurate representation of the demographics of students enrolled in the program for the past 4 years.

The pre-requisite first-year course ‘Cell Biology’ was required prior to enrolment in the histology course, students were also required to enrol in ‘Human Physiology’ in the same semester. To some extent students had histology knowledge prior to starting the course because of the pre-requisite and co-requisite courses. Therefore, to exclude any bias resulting from previous or current knowledge a pre-test quiz was conducted at the beginning of the course in both 2016 (*n* = 26) and 2017 (*n* = 28). The average mark for the pre-test result in 2016 was 77% (SD ± 13) and in 2017 was 74% (SD ± 22) and were not statistically different (*P* value 0.58). The pre-test result was also analysed for bias between the 2017 ‘video’ and ‘control’ group and was also not statistically significant (*P* value 0.15).

### Analysis of in-class and online use of learning material

**Fig. 3:**
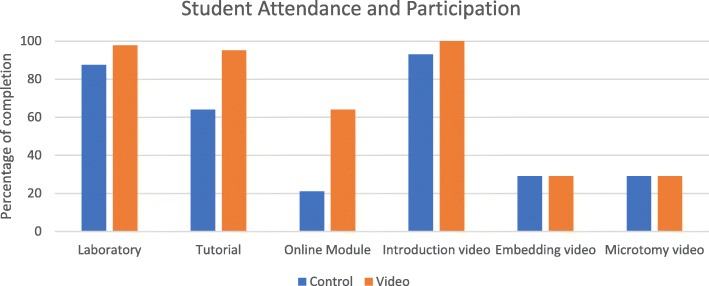
A comparison of frequency analytics of student participation in online and in-class learning activities for students in the control and video groups

All students were given access to the same in-class laboratory and online learning content throughout the course. All activities were voluntary except for a requirement to attend at least 80% of laboratory practicals. Frequency analytics of the 2017 student cohort in-class attendance (laboratory and tutorial), online material usage (interactive histology module) and viewed recordings of expert demonstrations of histology equipment (introduction to the histology laboratory; embedding demonstration; and microtomy demonstration) are reported by percentage for each group: ‘video’ compared to ‘control’ in Fig. 3.

As there was a minimum 80% requirement for laboratory attendance, participation in the practical component of the course was high for both groups (control = 87.5%, video = 97.7%), as was viewing the six-minute ‘Introduction to Histology’ video (control = 93%, video = 100%), which was available in the laboratory practical, through the student’s online portfolio and in the online e-Learning module. In total, the ‘Introduction to Histology’ video was viewed 94 times. Total time spent on task was 3 h and 47 min (control = 1 h 17 m, video = 2 h 40 m) throughout the 13-week semester.

Differences in participation between the two groups related to the voluntary attendance at tutorials and the completion of the online e-Learning histology module. The tutorials were well attended by the video group (95.1%) in comparison to the control group (64.0%). From the 2017 cohort a half of the students completed the voluntary online e-Learning module and most of these students (77%, *N* = 10) were in the video group. In total, the module was completed 29 times, total time spent on task was 6 h and 48 min (control = 1 h 21 m, video =5 h 27 m) and was repeated more than once by seven students in the video group. The average score was 86% (range 43–100%) and the average time spent on the lesson was 21 min (range 4–47 min). The eight-minute ‘Histology Technique’ videos made by the Instructor that depicted the ‘expert’ completing the embedding (4 min) and microtomy (4 min) techniques was viewed 25 times, by only eight students (control = 4; video = 4). Total time spent on task was 1 h and 30 min, with equivalent time on task recorded for both groups (control = 0 h 44 m, video = 0 h 46 m). Those students who viewed the expert video demonstrations of histology skills and completed the online histology module had an improved statistically significant final grade. Results of these assessments are reported in Table 2.

**Table 2:** Final grade results for the student cohort in 2017. Students who viewed the expert histology technique demonstration videos or completed the online e-Learning module had a significant increase in final grade compared to those students who did not

Online Tool	Participation (hours:minutes)	Final Grade %	*T* value	*P* value
Embedding video (4 min)	Viewed (*n* = 8) (control = 0 h 20 m; video = 0 h 15 m)	74.5 (± 7.27)	2.059	0.05
	Did not view (*n* = 20)	62.03 (± 24.54)		
Microtomy video (4 min)	Viewed (*n* = 8) (control = 0 h 24 m; video = 0 h 31 m)	75.25 (± 6.54)	2.279	0.03
	Did not view (*n* = 20)	61.72 (± 24.44)		
e-Learning module (21 min)	Completed (*n* = 14) (control = 1 h 21 m; video = 5 h 27 m)	76.7 (± 11.44)	2.838	0.01
	Did not complete (*n* = 14)	57.25 (± 24.03)		

### Quantitative analysis of the practical examination and the final grade

Students who participated in the video recording of the histology techniques (video group) in 2017 had an improved summative practical assessment (82.4%, ± SD 8.66) and showed a significant increase in final grade (75.6%, ± SD 12.74), compared to those in the ‘control’ group (55.6% ± SD 24.46; *P* value 0.01).

**Fig. 4:**
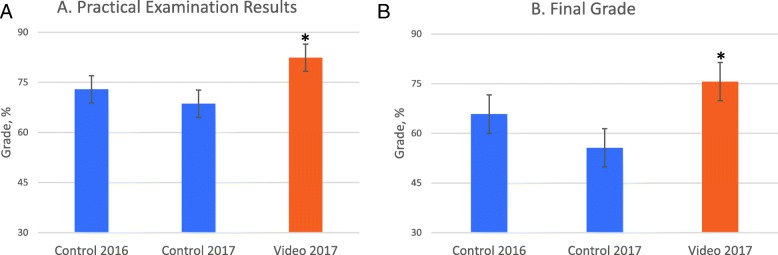
Assessment results by class cohort, ‘control’ versus ‘video’ groups. **a** Represents statistically significant results for the practical examination. **b** Represents statistically significant results for the final grade. Error bars represent standard error of the mean and *denotes a statistically significant result, *P* < 0.05

In comparison to the 2016 cohort, the 2017 ‘video’ group significantly improved on average by 9.5% for the practical assessment (*P* value 0.03) and 9.8% for the final grade (*P* value 0.03). The statistically significant summative practical assessment and final grade results are presented in Fig. 4.

### Student survey: Likert responses

Of the 28 students in the study, 19 (68% response rate) participated in the end of semester survey (control, *N* = 6; video, *N* = 13). This was the first time these students had enrolled in a course that utilised video feedback or expert video demonstrations for a practical component. Table 3 presents the student ratings of the statements on the use of video recording and feedback for histology procedures presented by mean and standard deviation using the 5 point Likert scale where 1 = strongly disagree to 5 = strongly agree. There were four main themes: (1) self-evaluation and improvement; (2) feedback; (3) peer learning; and (4) future use. When ‘video’ students were asked to assess their histology technique after viewing themselves by video, approximately 75% agreed or strongly agreed (*M* = 4.23, SD =1.23) that they could do so. Three quarters of the ‘video’ students agreed or strongly believed the video recording provided an authentic picture of their histology skills (*M* = 4.0, SD =1.47) and approximately two thirds strongly agreed (*M* = 3.77, SD =1.9) that they could make improvements after viewing their attempts.

**Table 3:** Mean and standard deviation for respondents rating their experience of video recording and feedback for histology procedures using the 5 point Likert scale where 1 = strongly disagree to 5 = strongly agree

Students rating responses		
	Mean	Standard Deviation
(1) Student evaluation and Improvement ‘Video’ group only (*n* = 13)		
The video recording allowed me to self-reflect on my histology technique	4.23	1.23
The experience of being recorded is valuable even if feedback from the Instructor is not provided	3.31	1.65
The video recording provided an authentic picture of my histology skills	4.0	1.47
My self-perception of how I completed the activity was different when I watched the video recording	3.53	1.51
After watching the video recording I learnt from my mistakes and felt that I could improve my histology technique	3.77	1.9
(2) Feedback ‘Video’ group only (n = 13)		
The video recording allowed me to receive alternative feedback that enhanced my learning	4.15	1.21
It was necessary that I received feedback from the Instructor that accompanied the video recording	3.92	1.44
(3) Peer learning ‘Video’ and ‘control’ groups (*n* = 19)		
My learning would be enhanced if I could watch the recordings of my peers	3.34	1.61
Students should provide feedback to other peers after viewing the recorded video	3.05	1.43
(4) Future use ‘Video’ and ‘control’ groups (*n* = 19)		
I would use the expert and/or my video to study before for the practical exam	3.53	1.71
I would use the expert and/or my video for learning after completing the histology course (e.g. before I attend placement or as a graduate)	3.47	1.68

Three quarters of the students agreed or strongly agreed (*M* = 4.15, SD =1.21) that video feedback was an alternative way to receive feedback that enhanced learning, and two thirds agreed or strongly agreed (*M* = 3.92, SD =1.44) that individual feedback provided by the assessor while watching the video, was also necessary.

Responses to peer learning was variable and indicates students are ‘unsure’ if peer feedback would benefit learning (*M* = 3.05, SD =1.43). While some students believed peer learning would strongly enhance their knowledge and skill acquisition (33.3%) and peers should provide feedback (16.7%), other students strongly disagreed with peers providing feedback (25%) and thought that this process would not improve knowledge, and learning would not be enhanced. Approximately two thirds of the respondents agreed or strongly agreed (*M* = 3.53, SD =1.71) that they would use a video recording (either their own or from the expert) for study purposes and agreed they would use the video recording again after completion of the course or as a revision tool during placement in the profession (*M* = 3.47, SD =1.68).

### Qualitative survey: open-ended responses

**Fig. 5:**
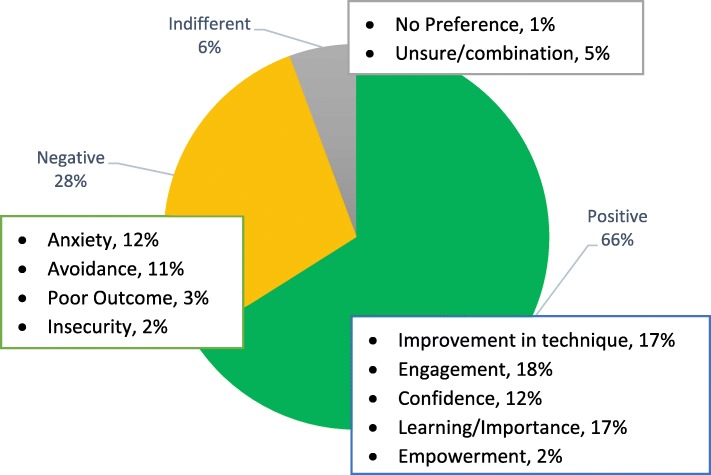
Percentages of positive, negative and indifferent comments from respondents to the open-ended survey data of student’s perspective of video recordings

The open-ended question data from the survey identified whether respondents had a positive, negative or indifferent experience concerning the video recordings. Respondent answers were analysed to identify a theme or keyword that was used in a coding scheme [11] by two academic experts. The three themes included comments that were: (1) positive - improvement in technique, engagement, confidence, learning/importance and empowerment; (2) negative - anxiety, avoidance, poor outcome, insecurity; and (3) indifferent - no preference, unsure, combination of positive and negative comments. A summary of the participants responses is presented in Fig. 5.

Two academics coded 115 open-ended responses from the 19 participants with an intraclass correlation coefficient of 0.924 and Cronbach’s Alpha 0.918, suggesting excellent agreement between raters. The majority (66%) expressed positive comments which highlighted engagement, improvement in technique and learning importance. A typical response was: “*I was grateful for the opportunity to have a video made of my attempt at embedding tissue ready for microtomy. By viewing and receiving timely feedback of my video, I was able to improve my embedding technique and confidence level*”. Most students did not find the recording device intrusive or uncomfortable and some “*forgot about being recorded*” once they began the histology technique and the experience was positive “*as it helped with exam nerves knowing I have been watched closely before [through video]”.* The negative comments (28%) involved anxiety of being recorded *“I felt nervous being recorded”* and avoidance, not wanting to participate or complete the activity while being recorded in front of other peers *“I would not recommend being recorded, I only want verbal or written feedback at the time of my attempt”.* A small proportion of responses (6%) were indifferent such as: *“I’m not sure if the videos are helpful, or if I learnt from them”.*

## Discussion

The goals of this study were to investigate whether video feedback and online resources in a MLS program provided value and engagement in terms of student performance and perceptions; and how video and online assisted feedback could be designed to promote interactive and engaging experiences.

In regard to student performance this study found two things. Firstly, improved exam scores and secondly improved engagement for the ‘video’ group. For example, there were significant differences in learning outcomes between the control and video group experiences. Average final grade for the 2016 ‘control’ group was 65.83%, (±SD 18.86) compared to the ‘video’ group 75.6%, (±SD 12.74). Secondly, there was an increase in voluntary engagement for the ‘video’ group compared to the 2017 ‘control’ group through tutorial attendance (control = 64%; video = 95%), ‘Introduction to Histology’ video views (control = 1 h 17 m; video = 2 h 40 m) and time on task for the e-Learning module (control = 1 h 21 m; video =5 h 27 m).

These findings are consistent with other studies that have found comparable or improved gains using video feedback [8, 12, 13]. The literature in video feedback reports themes in ‘nervousness’ of being video-recorded with subsequent ‘reflective dialogue that increases self-knowledge’ [8] and a high level of satisfaction with individual feedback provided by the Instructor [13, 14]. Videos demonstrated by the expert have ‘helped recreate the in-person laboratory session’ experience [12] and watching one’s own performance through video improved confidence, clinical knowledge and learning [13], which was similarly reported in the ‘video’ group.

E-Learning and interactive virtual learning benefits for the student are mixed. Literature reports improved gains through increased exam scores [15, 16], flexibility to learn anywhere and anytime [17] and positive attitudes towards virtual experiences [18]. However, comparable attitudes and performance to the traditional face-to-face instruction [19], or poorer results [4] have also been reported in the health sciences education literature.

### Motivation to learn

It was important to not only assess improved grade outcomes but also to analyse the students’ perspectives and self-perceptions of their engagement and learning experiences. After implementing any curriculum change using online learning it is important to ask, “do students use the feedback to improve and what makes students pay attention to feedback?”. These questions underpin achievement goal theory, the motivation behind the learners’ decisions and behaviours to achieve a learning goal [20, 21] or in essence the motivation to learn.

We analysed how often and when the students used the online material for knowledge acquisition, revision or feedback. Moreover, the qualitative analysis provided an in-depth response to motivation and engagement. Whilst it was shown that video-assisted feedback and online learning improved grade outcomes, not all students liked being video recorded, stated anxiety, or were not motivated to learn using online resources. It has been reported that feeling challenged whilst learning may enhance learning, and some have argued that this emotional response could heighten attention to the activity and improve learning outcomes [22]. Challenging students within their comfort zone may improve learning but over challenging may have the opposite effect by causing anxiety or avoidance. Anxiety in learning is a pathway to situational interest, “situational interest is triggered by the complex interplay between the student’s interest and goals and the teaching environment” [23]. Moderate levels of anxiety are motivating, triggering students to work harder and this may explain why those students allocated to the ‘video’ group had increased time on task and engaged more than the control group in both online and face-to-face learning activities. The perceived ‘anxiety’ of being video recorded while completing a task and having an ‘expert’ analyse the technique may promote learning attributes such as participation and engagement in voluntary learning activities. For the control group the anxiety is less, and the task is seen as unimportant which may lead to reduced interest and engagement.

### Feedback

The effect of a known interaction which ‘makes you focus because you will be scrutinised’ is part of the social system of learning that can readily engage students in an online environment [24]. Understanding how students prepare and their motivation to use learning resources and seek feedback is important when designing learning activities that are online or simulated [25]. However, previous research has shown that perceived versus actual online engagement is full of contradictions and merely offering online resources with feedback is not necessarily sufficient to promote learning [26, 27]. Both the video and control group were offered the same online resources however, the video group engaged up to four times more than the control group as evidenced by time on task (e-Learning module control = 1 h 21 min; video = 5 h 22 min).

While motivation and emotion have been recognized as factors that influence how students interpret and use feedback [25, 28] the learning style and the nature of the task are also influencing factors [29]. Performance-oriented goals, whereby students want positive affirmation of their proficiency when performing a learning activity which thereby focusses their motivation to achieve [20] was notable in the video group. The nature of the task, being assessed by an Instructor, influenced this group to be motivated to achieve, this group had a much richer experience by choosing to increase their exposure to the content. The learning style ‘to master the technique’ *before* being assessed by an Instructor is likely a strong influencing factor to perform well, compared to being assessed by a peer. The control group who received feedback by their peers performing the formative histology techniques did not have the same motivation to engage in the online resources, and therefore had less exposure to content. As evidenced by the reduced time on task and tutorial participation (tutorial attendance control = 64%; video = 95%) the control group did not have the same performance-oriented goals.

Moreover, achievement goals have been shown to influence feedback-seeking behaviours. In a meta-analysis of the goal orientation literature, Payne et al. [30] found that feedback-seeking was positively related to mastery-approach goals such as ‘hands-on’ skills. The ‘educational alliance’ and the importance of instructor presence (whether online or face-to-face) to foster a feedback relationship and the learners’ perceptions of the supervisory relationship is also important [31]. The ‘anxiety’ response and the need to be ‘prepared’ for Instructor feedback may explain why the video group spent more time on task. Video assisted feedback provides objective evidence of an individual’s performance because it provides accurate real-time data and can be viewed multiple times [13], there is no place to ‘hide’ or recollect a different perception of the activity.

Most students reported that their “*self-perception of completing the histology activity was different compared to the video recording*” (M = 3.53, SD = 1.51) however, after watching the video and receiving feedback from the ‘expert’ they agreed that “*it provided an authentic picture of their histology skills*” (M = 4.0, SD = 1.47). Agreeable student responses such as: “*the video recording allowed me to receive alternative feedback that enhanced my learning”* (M = 4.15, SD = 1.21) and “*it was necessary that I received verbal/written feedback from the Instructor that accompanied the video recording*” (M = 3.92, SD = 1.44) highlights the importance of prompt and accurate feedback to provide an authentic picture of the performance however, these statements do not reflect the associated time and effort taken by the assessor to provide the feedback.

Although the ‘video’ group consisted of only 14 students and the total time for providing feedback was approximately three and a half hours (5 min per student to provide oral feedback and approximately 10 min for video review and written feedback) this level of feedback could not be replicated in a larger cohort. It could be suggested that students peer review themselves to reduce the marking and feedback time associated with formative review. Albeit, this will likely change the engagement and motivation associated with the achievement goal and feedback-seeking behaviours as was experienced in the 2017 ‘control’ group in this study.

Interestingly, no students in this study reported anxiety or avoidance when watching the ‘expert’ video technique or when receiving feedback from a peer. In fact, a quarter of students believed peer feedback did not enhance learning and over a half (58%) would not recommend peer-feedback for learning a histology technique. The usefulness of student feedback reported in the literature is mixed. Some studies strongly believe peer review benefits learning and critical thinking [30, 32]while improving the capacity to provide feedback in large courses [33], while others do not find it useful or report avoidance behaviours to not participate in peer feedback [34]. Additionally, self-evaluations have been reported to be more specific and useful than peer-feedback [35] but it could be argued that this would mitigate the importance of Instructor presence and the perceived social benefit of being reviewed by the ‘expert’. Alternatively, in review of the time commitment for the assessor to provide feedback for a larger cohort, it may be plausible for all students to video record their performance and the Instructor randomly selects some students to be assessed. This may maintain engagement and motivation for performance-oriented goals and encourage the use of learning resources and feedback seeking behaviours.

The motivation behind the learners’ decisions and behaviours to achieve a learning goal is multi-faceted and is likely influenced by many explicit and implicit variables. This study reinforces the importance of motivation, emotion and the social nature of learning. In particular, carefully designed and evaluated blended learning tools can have both direct and indirect effects on final grades by improving knowledge and increasing engagement in the course [36]. Studies indicate that noncognitive skills, such as motivation and conscientiousness, are crucial factors in the efficient development of cognitive skills in science learning [37]. It has been reported from the same authors that online simulations can increase both learning outcomes and motivation levels. However, the final stage in learning requires real equipment that must be acquired with hands-on experience and expertise in learning [38].

## Conclusions

It is not anticipated that online learning will completely replace the physical presence and hands-on learning in the laboratory. Nonetheless, our study shows that a blended learning approach that combines traditional hands-on learning with educational technology enhances learning in the laboratory and has benefits for both the student and the academic. Virtual or online learning experiences may be effective alternatives when the hands-on approach is too complex for early learners, expensive, or inaccessible due to laboratory constraints, or the activity is too time consuming to complete in the laboratory. Developing online resources that simulate and prepare the student for laboratory procedures promotes an enriched and sustainable learning experience. The student is more likely to be engaged and motivated to learn because they have attempted the simulated procedure and had multiple opportunities to learn the procedure prior to the laboratory session. Specifically, video and online assisted feedback provided by the *expert* improves grade outcomes and improves engagement through motivation which is linked to achievement goal theory.

Limitations to adopting this combination of learning methods include the educational technology expertise to develop the online tools, the time allocated to feedback and developing such technologies, and the necessary motivation and engagement of the student to accept and adopt the blending learning approach to their studies. It is plausible that the conscientious student who readily engages with blended learning or has previous experience in online learning or video feedback will perform better regardless of the learning intervention. Without strong financial and institutional support, it is difficult to achieve positive outcomes when introducing blended learning to curriculum renewal in medical science programs.

## Data Availability

The datasets used and/or analysed during the current study are available from the corresponding author on reasonable request.
